# Circulating exosomal microRNA expression patterns distinguish cardiac sarcoidosis from myocardial ischemia

**DOI:** 10.1371/journal.pone.0246083

**Published:** 2021-01-26

**Authors:** Elliott D. Crouser, Mark W. Julian, Sabahattin Bicer, Vikas Ghai, Taek-Kyun Kim, Lisa A. Maier, May Gillespie, Nabeel Y. Hamzeh, Kai Wang

**Affiliations:** 1 Department of Internal Medicine, Division of Pulmonary, Critical Care and Sleep Medicine, The Dorothy M. Davis Heart and Lung Research Institute, The Ohio State University Wexner Medical Center, Columbus, Ohio, United States of America; 2 Institute for Systems Biology, Seattle, Washington, United States of America; 3 Department of Medicine, Division of Environmental and Occupational Health Sciences, National Jewish Health, Denver, Colorado, United States of America; 4 Department of Internal Medicine, Division of Pulmonary, Critical Care and Occupational Medicine, University of Iowa Carver College of Medicine, Iowa City, Iowa, United States of America; Kunming University of Science and Technology, CHINA

## Abstract

**Objective:**

Cardiac sarcoidosis is difficult to diagnose, often requiring expensive and inconvenient advanced imaging techniques. Circulating exosomes contain genetic material, such as microRNA (miRNA), that are derived from diseased tissues and may serve as potential disease-specific biomarkers. We thus sought to determine whether circulating exosome-derived miRNA expression patterns would distinguish cardiac sarcoidosis (CS) from acute myocardial infarction (AMI).

**Methods:**

Plasma and serum samples conforming to CS, AMI or disease-free controls were procured from the Biologic Specimen and Data Repository Information Coordinating Center repository and National Jewish Health. Next generation sequencing (NGS) was performed on exosome-derived total RNA (n = 10 for each group), and miRNA expression levels were compared after normalization using housekeeping miRNA. Quality assurance measures excluded poor quality RNA samples. Differentially expressed (DE) miRNA patterns, based upon >2-fold change (*p* < 0.01), were established in CS compared to controls, and in CS compared to AMI. Relative expression of several DE-miRNA were validated by qRT-PCR.

**Results:**

Despite the advanced age of the stored samples (~5–30 years), the quality of the exosome-derived miRNA was intact in ~88% of samples. Comparing plasma exosomal miRNA in CS versus controls, NGS yielded 18 DE transcripts (12 up-regulated, 6 down-regulated), including miRNA previously implicated in mechanisms of myocardial injury (miR-92, miR-21) and immune responses (miR-618, miR-27a). NGS further yielded 52 DE miRNA in serum exosomes from CS versus AMI: 5 up-regulated in CS; 47 up-regulated in AMI, including transcripts previously detected in AMI patients (miR-1-1, miR-133a, miR-208b, miR-423, miR-499). Five miRNAs with increased DE in CS included two isoforms of miR-624 and miR-144, previously reported as markers of cardiomyopathy.

**Conclusions:**

MiRNA patterns of exosomes derived from CS and AMI patients are distinct, suggesting that circulating exosomal miRNA patterns could serve as disease biomarkers. Further studies are required to establish their specificity relative to other cardiac disorders.

## Introduction

Sarcoidosis is an idiopathic granulomatous disorder that most often involves the lungs; however, cardiac complications of sarcoidosis are common and potentially life-threatening [1–3]. As recently as 20 years ago, the prevalence of cardiac involvement among patients with sarcoidosis was estimated to be <5%. Since the advent of advanced cardiac imaging techniques, such as cardiac magnetic resonance and positron emission tomography, the prevalence of cardiac sarcoidosis (CS) is now estimated to be ~25%, which is in keeping with autopsy studies [1]. However, the routine use of advanced imaging studies for the detection and monitoring of CS is expensive, inconvenient, and carries some risk (e.g., radiation exposure during PET scan). As such, there is an urgent need to develop a blood-based circulating biomarker for CS detection.

Recent studies have reported that microvesicles released from diseased cells can be used to detect and monitor the progression of disease. More specifically, small lipid vesicles (30–100 nm diameter) containing genetic cargo from the parent cell, referred to as exosomes, can be detected in the circulation to identify various diseases, ranging from several different cancers [4] to Alzheimer’s disease [5]. Microvesicles are abundant in the plasma, and these include relatively large fragments of damaged cells and smaller exosomes that arise from endosomes containing cell specific genetic and protein cargo. Exosomes arising from diseased cells often carry a unique pattern of molecules including non-coding RNAs and proteins, which can be leveraged as biomarkers for disease diagnosis including heart disease. We previously showed that CS patients have distinct circulating exosomal microRNA (miRNA) expression patterns compared to sarcoidosis patients with no cardiac involvement [6].

CS often presents with chest pain and/or arrhythmias, clinical features shared with acute myocardial infarction (AMI) [7–10], including relief of chest pain with nitrates [7], leading to an expensive diagnostic evaluation for an ischemic etiology. In view of the need for better biomarkers, we sought to determine whether non-coding miRNA expression patterns in circulating exosomes could distinguish CS from AMI. We analyzed blood samples that were obtained from prior National Institutes of Health (NIH)-sponsored clinical trials/studies. Despite variables relating to blood processing and duration of storage, the study validated previously reported circulating miRNA markers of AMI and identified some potential novel biomarkers for CS.

## Materials and methods

### Study samples

As existing, de-identified samples were received from their approved holders for further analysis, this study was determined to require no further review nor approval by The Ohio State University Biomedical Sciences Institutional Review Board (# 2017E0627). We conducted a retrospective study of 40 patients (20 discovery and 20 validation) with histologically proven sarcoidosis who had clinical and radiographic evidence of CS, based upon established criteria [2, 11, 12], compared to 20 patients (10 discovery and 10 validation) having a history of AMI, and 20 age-matched, healthy, control subjects (10 discovery and 10 validation). Well-phenotyped, de-identified platelet-depleted plasma samples were collected within the scope of the NIH-funded A Case Controlled Etiologic Study of Sarcoidosis [(ACCESS), 13] research study from sarcoidosis case and matching controls; whereas, similarly characterized serum samples were obtained as part of the NIH-funded Genomic Research in Alpha-1 Antitrypsin Deficiency and Sarcoidosis [(GRADS), 12] and Thrombolysis in Myocardial Infarction [(TIMI II), 14] research studies. The ACCESS study was performed >20 years ago, and the plasma samples have been cryopreserved since that time. Likewise, the serum samples from the TIMI II trial and GRADS study have been similarly stored and kept frozen over the >30 years and ~5 years since they were completed, respectively. These samples were obtained from the NIH Biologic Specimen and Data Repository Information Coordinating Center (BioLINCC) repository and from National Jewish Health.

### Exosomal fraction isolation

Before exosome isolation, the plasma/serum samples were spun at 10,000 x *g* at 4°C for 10 minutes to remove cell debris. The exosomal fraction was then enriched from either 250 μl of clarified plasma or serum using the Total Exosome Isolation kits (for plasma or serum, respectively) (Life Technologies, Inc.; Carlsbad, CA), according to the manufacturer’s recommendations. The isolated exosomes were characterized with NanoSight (Malvern Panalytical, Ltd.; Malvern, UK) and by light and electron microscopy. Briefly, following isolation, exosomal pellets were re-suspended in 250 μl of PBS and examined using a Malvern NanoSight NS300 equipped with a 532 nm laser light-scattering (no labelling required) analysis to determine the size distribution and concentration of the isolated particles. Sample concentration was adjusted until a clear image was obtained of a population of around 100 particles in the scattering volume. Three videos of 60-second duration were taken with a frame rate of 30 frames/second, and particle movement was analyzed using Nanoparticle Tracking Analysis (NTA) software (version NTA 3.3, Dev Build 3.3.104; NanoSight, Ltd.; Salisbury, UK). NTA analyzes videos captured using the instrument, providing a particle size distribution and particle count based upon tracking of each particle's Brownian motion. Tracking is carried out for all particles in the laser-scattering volume to produce a particle size distribution using the Stokes-Einstein equation, relating the Brownian motion of a particle to a sphere-equivalent hydrodynamic radius.

For transmission electron microscopic (TEM) visualization, 50 μl exosomal solution in PBS was fixed with 500 μl of 2% paraformaldehyde and glutaraldehyde solution. After overnight fixation at 4°C, exosomes were carefully loaded onto charged formvar-coated copper grids for 10 minutes, then grids were stained with 2% uranyl acetate for 1 minute, rinsed, blotted dry and imaged at 80 kV using the FEI Tecnai™ G^2^ Spirit TWIN TEM (FEI Company; Hillsboro, OR).

### RNA extraction, library construction and miRNA expression

Total RNA was extracted from the exosomal fractions using the Total Exosome RNA and Protein Isolation kit (Life Technologies, Inc.) per the manufacturer’s recommendations. The RNA was then eluted in nuclease-free water followed by quantity and quality assessment using the Agilent 2100 Bioanalyzer (Agilent Technologies; Santa Clara, CA) and the NanoDrop 1000 Spectrophotometer (Thermo Scientific; Wilmington, DE). To identify potential miRNA biomarkers, we assessed the miRNA spectrum in the discovery samples using next-generation sequencing (NGS). Small RNA sequencing libraries were constructed using the NEBNext^®^ Small RNA Library Prep set (New England BioLabs; Ipswich, MA) and run on NextSeq™ 500/550 High Output kits v2.5 (Illumina; San Diego, CA) with single-end 50-nucleotide read-length. Given the variable ages of the samples (~5–30 years), quality assurance measures were taken (principal component analysis, Spearman correlation of mapped reads, read numbers >1 million per sample) to assure adequate RNA quality. Data processing and miRNA mapping were performed as described previously [15] with subsequent miRNA expression internally normalized using read count per million (RPM) of processed read and then log2 transformed. Differential expression analysis was performed by edgeR Bioconductor package on samples with more than 1 million reads [16, 17]. To be considered to have a significant change in expression, a miRNA required >2.0 fold-change (or >1.0 log2 fold-change) with a p-value < 0.01 (calculated using the Wilcoxon rank sum test). Statistical comparisons of differentially expressed (DE) miRNAs (identified when comparing the Control and CS plasma groups or the AMI and CS serum groups) were corrected for multiple hypothesis testing based upon the Benjamini-Hochberg procedure to reduce false discovery [18]. NGS results for specific potential biomarker miRNAs were confirmed on the corresponding validation samples by employing Quantitative Reverse Transcription Polymerase Chain Reaction (qRT-PCR) performed using TaqMan Advanced miRNA assays (Thermo Fisher; Waltham, MA). The level of miR-16-5p was used for normalization, as it was an invariant miRNA in the sample set (low coefficient of variance across samples) based upon the miRNA mapping, as previously described [15]. Relative miRNA concentrations are presented by ΔCt (cycle threshold) values (Ct_reference_—Ct_target_) [19]. The biological processes impacted by the DE miRNAs were determined by using the MicroRNA Enrichment Analysis and Annotation (miEAA) tool [20].

### Statistics

The data were expressed as mean ± SEM, and statistical significance was based upon a value of p ≤ 0.05. SigmaPlot 14.0 and SYSTAT 13.0 software were used to plot the data and carry out the analyses, respectively. The Mann-Whitney U-test (Wilcoxon Rank Sum) was employed to compare group differences in qRT-PCR-validated samples between CS and control plasma exosomal transcripts and between CS and AMI serum exosomal transcripts. In addition, the U-test was used to compare the mean particle size at the distribution peak, the total concentration yield and the dynamic light scatter results from the NanoSight analyses of the extracted exosomes for each comparative pair.

## Results and discussion

### Study subjects

Patient demographics are shown in Table 1 based upon the plasma and serum samples conforming to CS, AMI or age-matched, disease-free controls obtained as part of the previously identified NIH-funded research studies with no significant differences between the compared groups.

**Table 1:** Patient demographics.

Samples	Sample Use	Groups	Age (years)	Gender (M/F)	Race (W/B/O)^a^
**ACCESS Study (Plasma)**	Discovery	Control (n = 10)	44.0 ± 4.8	4/6	7/3/0
	Validation	Control (n = 10)	43.0 ± 3.3	2/8	4/6/0
	Discovery	Cardiac Sarcoidosis (n = 10)	38.0 ± 3.7	5/5	6/4/0
	Validation	Cardiac Sarcoidosis (n = 10)	43.0 ± 2.9	5/5	6/4/0
**TIMI II Trial and GRADS Study (Serum)**	Discovery	AMI^b^ (n = 10)	51.8 ± 4.5	5/5	9/1/0
	Validation	AMI (n = 10)	51.4 ± 4.4	5/5	9/1/0
	Discovery	Cardiac Sarcoidosis (n = 10)	51.6 ± 3.4	6/4	7/3/0
	Validation	Cardiac Sarcoidosis (n = 10)	57.2 ± 3.5	7/3	9/0/1

^**a**^White/Black/Other

^b^AMI = Acute myocardial infarction

### Isolated exosomal fraction

**Fig 1:**
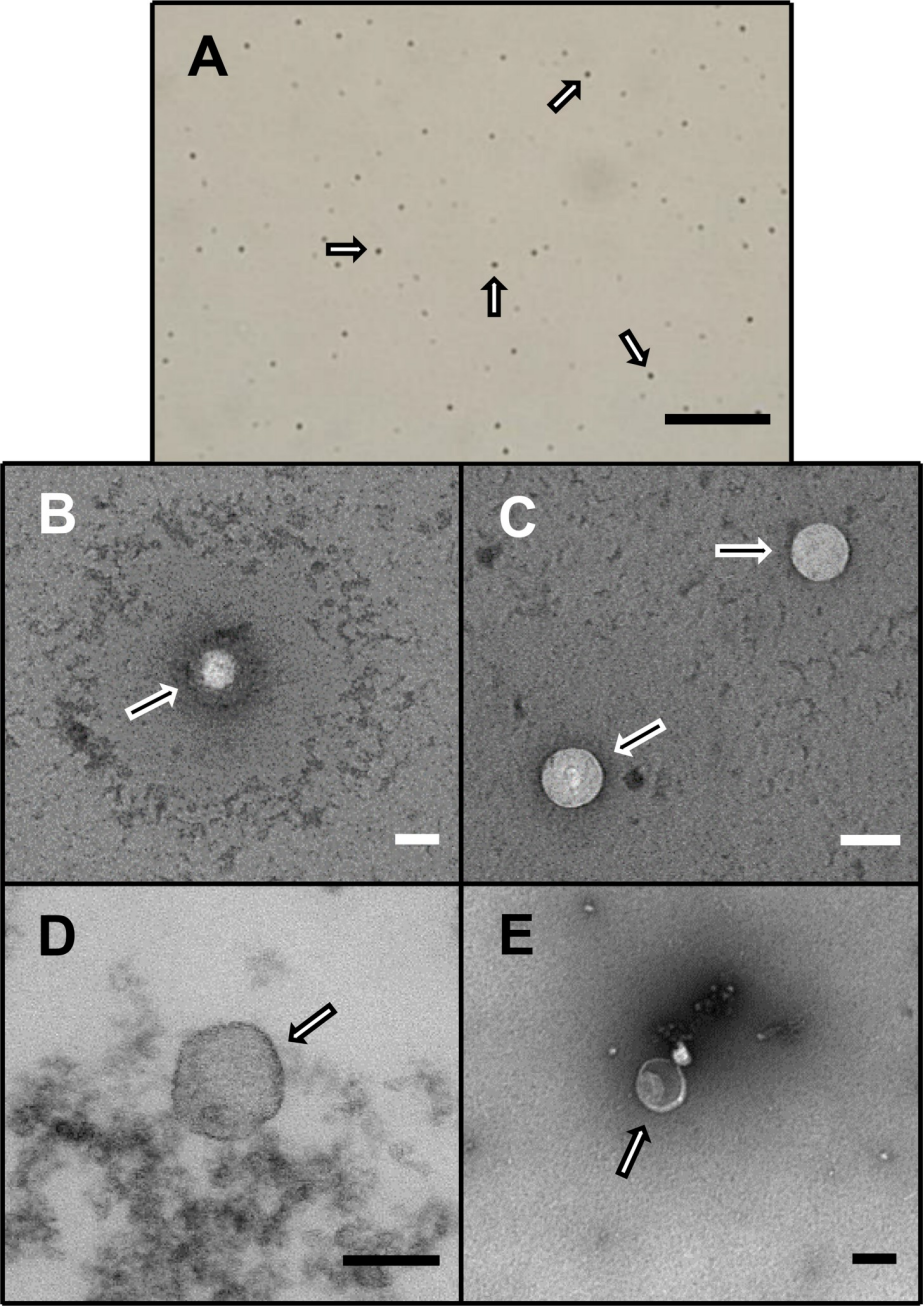
Exosomal visualization. Representative light (A) and electron (B-E) photomicrographs of exosomes, as marked by arrows, following isolation from plasma or serum samples from patients with cardiac sarcoidosis [scale = 1 mm (A) and 100 nm (B-E)].

Despite the extensive and varying age of the source samples and regardless of their being plasma or serum, exosomal isolation yielded surprisingly similar characteristic results suggesting that the exosomes, in general, remained remarkably intact while frozen over time and throughout the isolation process. Exosomes were relatively uniform in size and appearance across the different samples used in the study (Fig 1). Following NanoSight analyses, there were no apparent differences in the spectral profiles, size distribution, dynamic light scatter or concentration yield among the study groups (S1–S3 Figs).

### Sample quality assessment and miRNA detection

**Fig 2:**
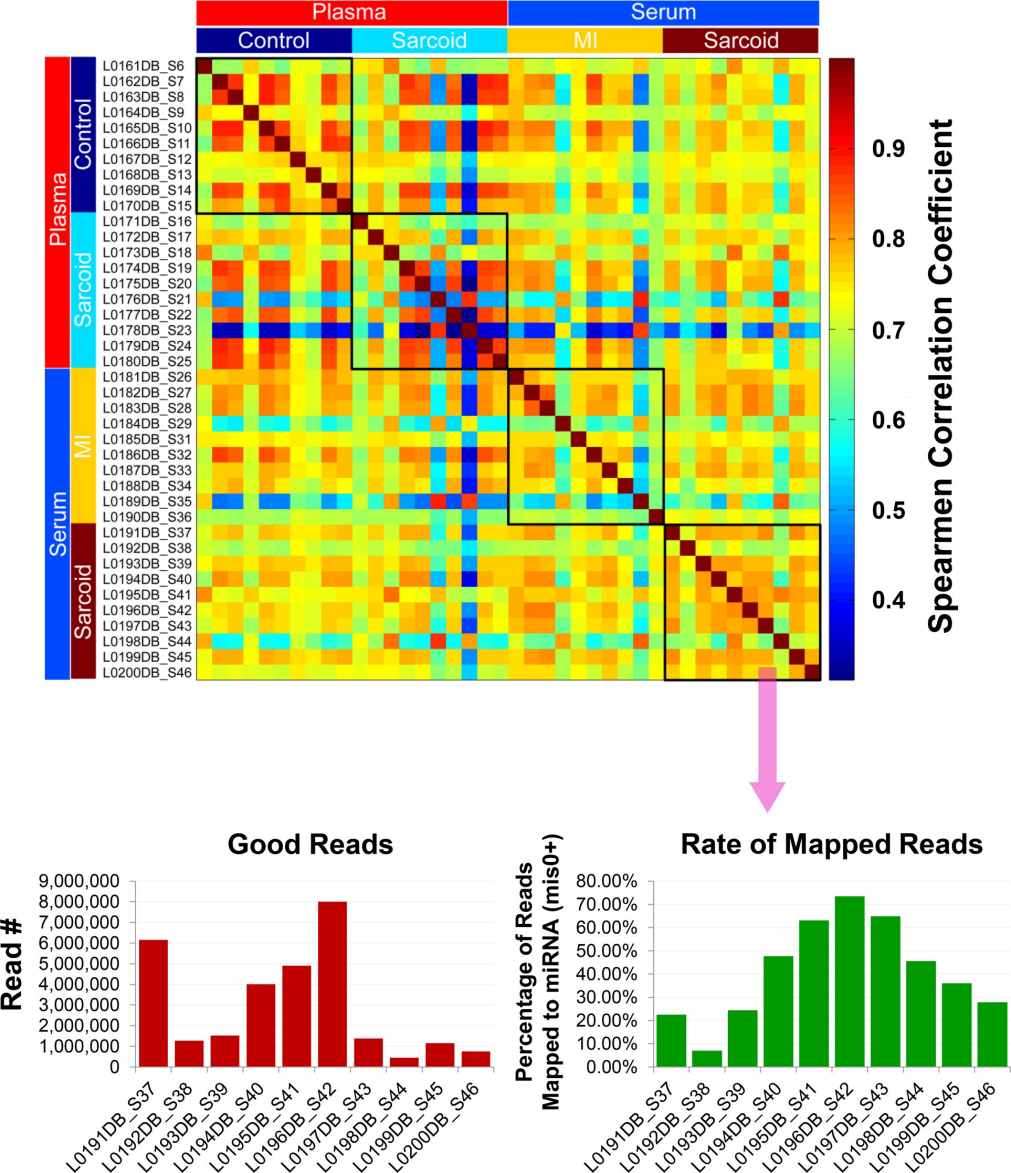
Exosomal miRNA quality determination. Quality assessment of exosomal miRNA isolated from the plasma/serum samples of the ACCESS/TIMI II/GRADS studies following next generation sequencing analyses. Spearman correlation analysis shows overall that the samples were highly correlated. Samples showing low correlation (blue) seemed to be related to the low read depth, which caused the reduced rate of mapped reads to miRNA for those particular samples (shown for cardiac sarcoidosis serum samples). Thus, samples with <10^6^ reads (5 total) were eliminated from the final differential expression analyses.

Given the advanced age of the samples (up to 30 years), further quality assurance measures were taken to identify and exclude inadequate and/or poor quality samples based upon gene expression. Principle component analysis (S4 Fig) and Spearman correlation coefficient analysis of mapped reads (Fig 2) demonstrated that the samples did not present any batch effect overall and that they were highly correlated. However, a few samples varied from the others and reflected a comparatively poor correlation due to low sequencing depth, causing a reduced rate of mapped reads to miRNAs (S4 Fig, Fig 2). Since this could lead to false positive results during DE analysis, 5 of the original 40 discovery samples were excluded, largely due to their having read numbers less than one million per sample [21, 22]. Although the overall quality of the RNA samples was relatively good despite the extensive and varied age of the samples, the number of miRNAs detected per sample varied with serum samples generally having fewer than in plasma, particularly when obtained from sarcoidosis patients (S5 Fig).

### DE miRNA identification and validation and resultant GO terms between comparative groups

**Fig 3:**
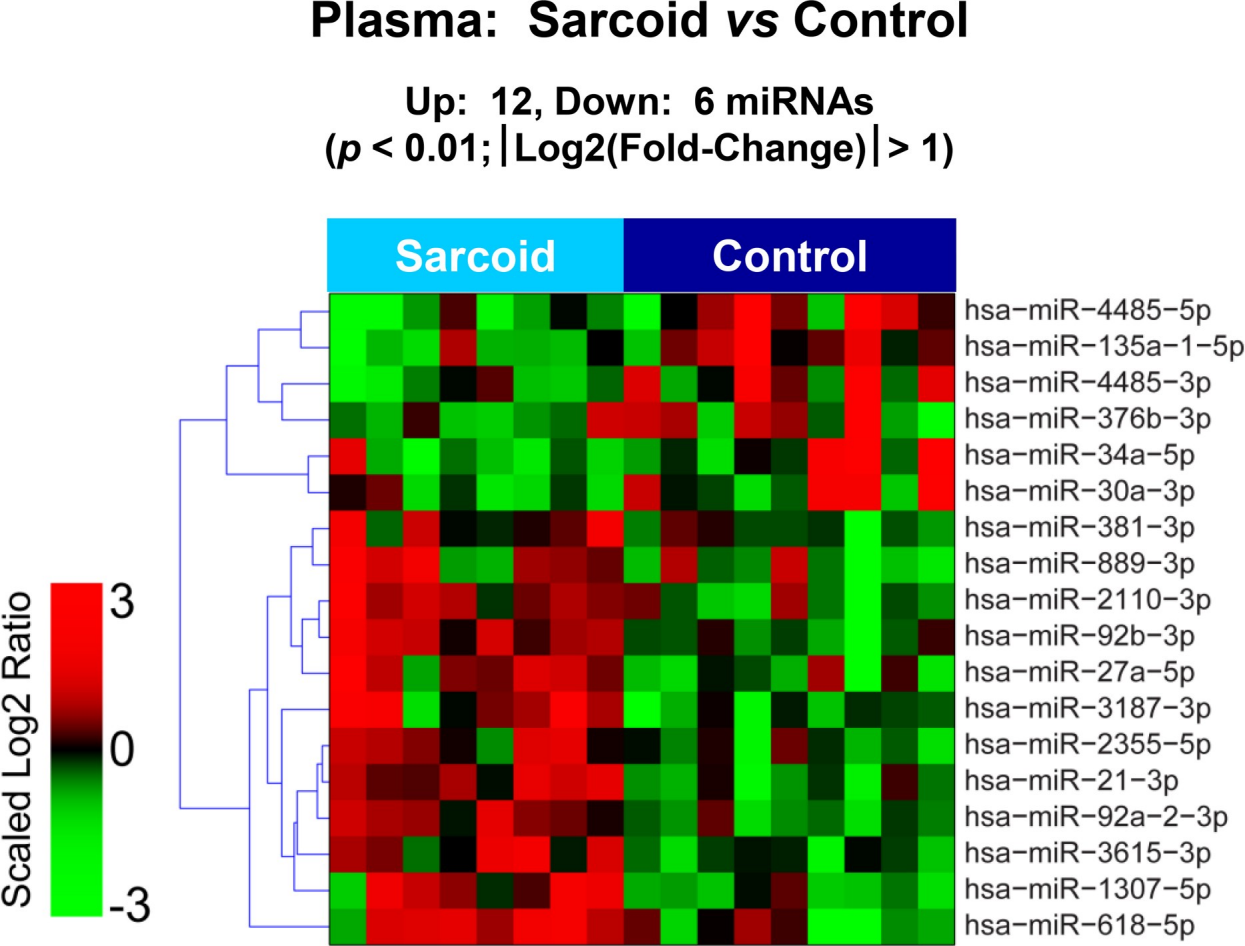
Plasma exosomal miRNA demonstrates differential expression between control and cardiac sarcoidosis. Heat map demonstrating exosomal miRNA differential expression following next generation sequencing analyses of control and cardiac sarcoidosis (CS) plasma samples. The results identified 12 up-regulated and 6 down-regulated significantly different miRs in CS relative to control [p < 0.01 and fold-change (FC) < 0.5 or > 2].

**Fig 4:**
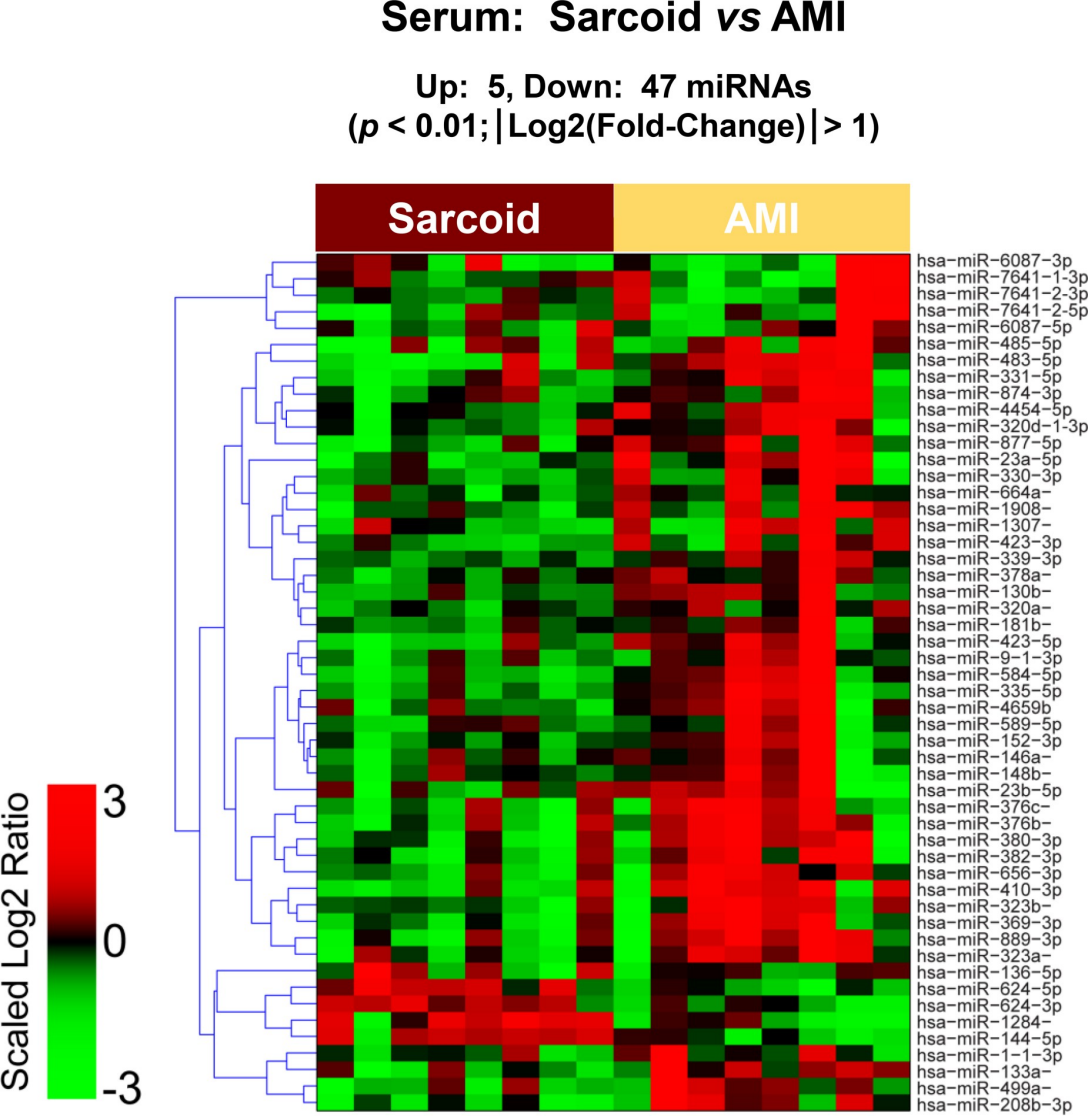
Serum exosomal miRNA demonstrates differential expression between cardiac sarcoidosis and acute myocardial infarction. Heat map demonstrating exosomal miRNA differential expression following next generation sequencing analyses of cardiac sarcoidosis (CS) and acute myocardial infarction (AMI) serum samples. The results identified 5 up-regulated and 47 down-regulated significantly different miRs in CS compared to AMI [p < 0.01 and fold-change (FC) < 0.5 or > 2].

**Fig 5:**
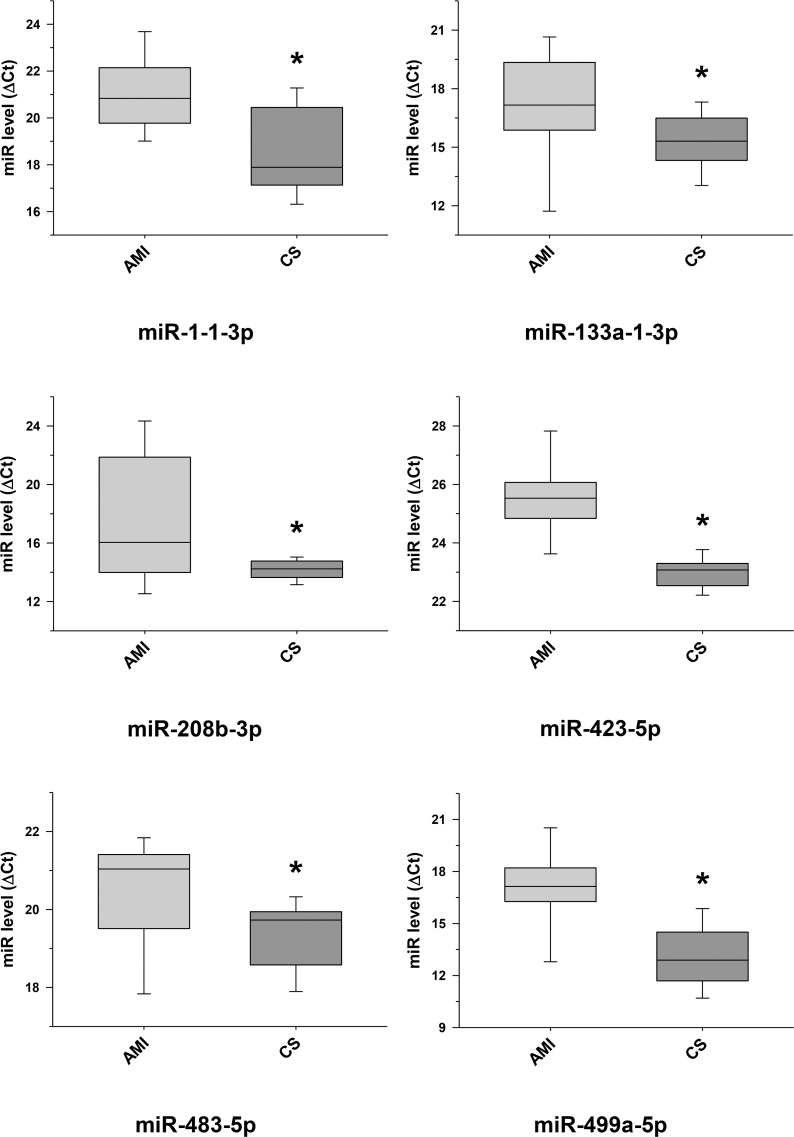
Validation of some selected differentially expressed miRNA transcripts between cardiac sarcoidosis and acute myocardial infarction serum exosomal samples. MiRNA transcripts, previously demonstrated and identified as markers of myocardial infarction and determined to be differentially expressed by next generation sequencing, were confirmed by qRT-PCR in the serum exosomal validation samples from the acute myocardial infarction (AMI) group and found to be expressed at significantly higher levels when compared to those of the cardiac sarcoidosis (CS) group (*p < 0.05).

**Fig 6:**
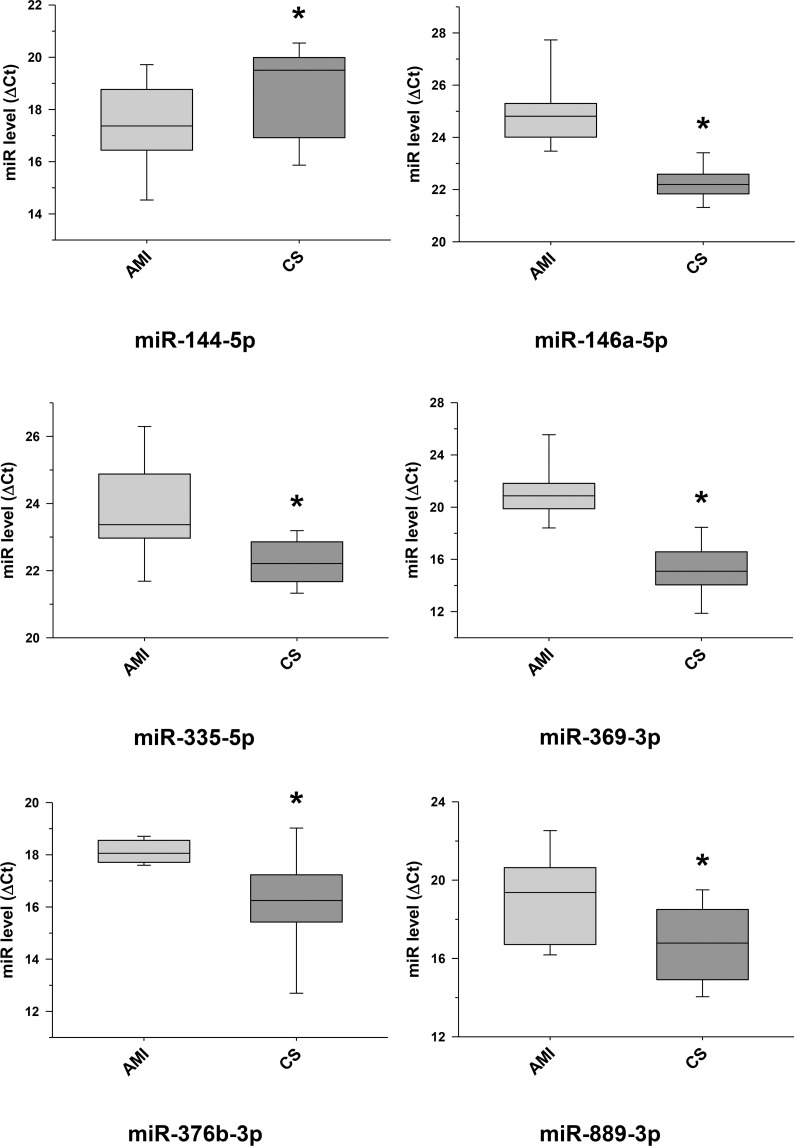
Further selected differentially expressed miRNA transcripts between cardiac sarcoidosis and acute myocardial infarction serum exosomal samples validated. Additional miRNA transcripts, determined to be differentially expressed by next generation sequencing when comparing the cardiac sarcoidosis (CS) and acute myocardial infarction (AMI) groups, were confirmed by qRT-PCR in the serum exosomal validation samples (*p < 0.05). MiR-144-5p, previously noted as a marker of cardiomyopathy, was validated to have a significantly higher level of expression in the CS group.

The raw sequence data have been deposited in NCBI's Sequence Read Archive and are accessible through BioProject accession number PRJNA674847 (https://www.ncbi.nlm.nih.gov/sra/PRJNA674847). For DE miRNA when comparing CS to control plasma exosomal samples, volcano plot (S6 Fig) and heat map (Fig 3) data presented 18 transcripts that were highly significant. Of those specifically identified (Table 2), miR-889-3p and miR-376b-3p were validated by qRT-PCR (S7 Fig). However, when comparing CS to AMI serum exosomal samples, volcano plot (S8 Fig) and heat map (Fig 4) data provided 52 DE miRNAs. Most of those exhibited higher concentrations in AMI (Table 3), of which 11 were validated by qRT-PCR (Figs 5 and 6). Many of these transcripts have been previously identified as biomarker candidates for myocardial infarction (Fig 5), substantiating the integrity of the TIMI II samples despite their long term frozen storage. GO term functional enrichment analysis of the DE miRNAs showing higher concentrations in AMI yielded considerable GO terms. Table 4 lists the most significantly affected functional terms, including a number of them associated with cardiovascular activities. Among the few identified as being more highly expressed in CS, miR-144-5p was validated (Fig 6). As expected, there were many DE miRNAs found when comparing plasma exosomal samples to serum exosomal samples in the CS group (S9 Fig).

**Table 2:** MiRNA that are significantly up- or down-regulated when comparing Cardiac Sarcoidosis (CS) to normal healthy controls.

miRNA Expression: CS > Control	Log2(Fold-change)	*p*-Value	miRNA Expression: Control > CS	Log2(Fold-change)	*p*-Value
hsa-miR-3187-3p	2.2	0.0006	hsa-miR-4485-5p	-3.5	0.0003
hsa-miR-27a-5p	1.8	0.0014	hsa-miR-34a-5p	-2.2	0.0029
hsa-miR-618-5p	1.7	0.0035	hsa-miR-135a-1-5p	-2.1	0.0010
hsa-miR-1307-5p	1.6	0.0001	hsa-miR-4485-3p	-1.9	0.0041
hsa-miR-21-3p	1.4	0.0006	hsa-miR-376b-3p	-1.8	0.0070
hsa-miR-3615-3p	1.4	0.0013	hsa-miR-30a-3p	-1.7	0.0043
hsa-miR-92b-3p	1.4	0.0027			
hsa-miR-2110-3p	1.4	0.0056			
hsa-miR-889-3p	1.3	0.0089			
hsa-miR-92a-2-3p	1.2	0.0022			
hsa-miR-381-3p	1.2	0.0096			
hsa-miR-2355-5p	1.1	0.0081			

**Table 3:** MiRNA that are significantly up- or down-regulated when comparing Cardiac Sarcoidosis (CS) to Acute Myocardial Infarction (AMI).

miRNA Expression: CS > AMI	Log2(Fold-Change)	*p*-Value	miRNA Expression: AMI > CS	Log2(Fold-Change)	*p*-Value
hsa-miR-136-5p	2.8	0.0030	hsa-miR-208b-3p	-5.1	3.5 x 10^−6^
hsa-miR-1284-5p	2.0	0.0008	hsa-miR-7641-2-5p	-3.9	0.0002
hsa-miR-624-5p	1.7	0.0035	hsa-miR-499a-5p	-3.8	3.0 x 10^−5^
hsa-miR-144-5p	1.4	0.0015	hsa-miR-6087-3p	-3.7	0.0014
hsa-miR-624-3p	1.4	0.0057	hsa-miR-584-5p	-3.2	6.2 x 10^−6^
			hsa-miR-23a-5p	-3.2	5.7 x 10^−5^
			hsa-miR-877-5p	-2.9	6.1 x 10^−5^
			hsa-miR-380-3p	-2.6	0.0014
			hsa-miR-423-5p	-2.6	4.3 x 10^−5^
			hsa-miR-889-3p	-2.5	0.0004
			hsa-miR-369-3p	-2.4	0.0001
			hsa-miR-376b-3p	-2.4	0.0008
			hsa-miR-485-5p	-2.3	0.0096
			hsa-miR-7641-1-3p	-2.3	0.0090
			hsa-miR-6087-5p	-2.2	0.0070
			hsa-miR-335-5p	-2.2	8.6 x 10^−5^
			hsa-miR-9-1-3p	-2.1	0.0011
			hsa-miR-423-3p	-2.0	0.0001
			hsa-miR-152-3p	-2.0	0.0007
			hsa-miR-133a-1-3p	-2.0	0.0011
			hsa-miR-410-3p	-2.0	0.0018
			hsa-miR-23b-5p	-2.0	0.0095
			hsa-miR-7641-2-3p	-2.0	0.0083
			hsa-miR-382-3p	-2.0	0.0025
			hsa-miR-330-3p	-1.9	0.0008
			hsa-miR-664a-5p	-1.9	0.0034
			hsa-miR-320d-1-3p	-1.9	0.0087
			hsa-miR-4659b-5p	-1.9	0.0052
			hsa-miR-323a-3p	-1.8	0.0042
			hsa-miR-323b-3p	-1.8	0.0037
			hsa-miR-376c-3p	-1.8	0.0023
			hsa-miR-1-1-3p	-1.8	0.0043
			hsa-miR-1908-5p	-1.8	0.0038
			hsa-miR-483-5p	-1.7	0.0037
			hsa-miR-148b-3p	-1.7	0.0051
			hsa-miR-874-3p	-1.7	0.0089
			hsa-miR-146a-5p	-1.7	0.0047
			hsa-miR-656-3p	-1.6	0.0068
			hsa-miR-4454-5p	-1.6	0.0031
			hsa-miR-589-5p	-1.5	0.0081
			hsa-miR-331-5p	-1.5	0.0075
			hsa-miR-1307-5p	-1.5	0.0063
			hsa-miR-181b-1-5p	-1.4	0.0064
			hsa-miR-130b-3p	-1.4	0.0019
			hsa-miR-320a-3p	-1.4	0.0030
			hsa-miR-339-3p	-1.2	0.0037
			hsa-miR-378a-3p	-1.2	0.0069

**Table 4:** Top 10 Gene Ontogeny (GO) biological processes enriched by down-regulated miRNAs (relevant when comparing cardiac sarcoidosis to acute myocardial infarction).

Subcategory	*p*-value	miRNAs/Precursors
**GO0045616**, regulation of keratinocyte differentiation	0.0000056	hsa-miR-146a-5p; hsa-miR-148b-3p; hsa-miR-378a-3p; hsa-miR-423-5p; hsa-miR-584-5p
**GO0047372**, acylglycerol lipase activity	0.000152	hsa-miR-148b-3p; hsa-miR-335-5p; hsa-miR-423-3p; hsa-miR-423-5p
**GO0055009**, atrial cardiac muscle tissue morphogenesis	0.000256	hsa-miR-148b-3p; hsa-miR-335-5p; hsa-miR-877-5p
**GO0060214**, endocardium formation	0.000256	hsa-miR-148b-3p; hsa-miR-335-5p; hsa-miR-877-5p
**GO0048845**, venous blood vessel morphogenesis	0.000267	hsa-miR-130b-3p; hsa-miR-148b-3p; hsa-miR-335-5p; hsa-miR-877-5p
**GO0046676**, negative regulation of insulin secretion	0.000316	hsa-miR-130b-3p; hsa-miR-148b-3p; hsa-miR-152-3p; hsa-miR-335-5p; hsa-miR-376b-3p; hsa-miR-376c-3p; hsa-miR-423-3p; hsa-miR-877-5p
**GO0031210**, phosphatidylcholine binding	0.000432	hsa-miR-130b-3p; hsa-miR-148b-3p; hsa-miR-335-5p; hsa-miR-378a-3p
**GO0051492**, regulation of stress fiber assembly	0.000432	hsa-miR-146a-5p; hsa-miR-148b-3p; hsa-miR-335-5p; hsa-miR-584-5p
**GO0010466**, negative regulation of peptidase activity	0.000623	hsa-miR-335-5p; hsa-miR-423-5p; hsa-miR-877-5p
**GO0043049**, otic placode formation	0.000623	hsa-miR-148b-3p; hsa-miR-335-5p; hsa-miR-877-5p

This study is the first, to our knowledge, to show that circulating exosomal miRNA expression patterns can distinguish AMI from CS. Despite the advanced age of many of the samples, exosomal miRNA expression patterns observed in AMI in this study are concordant with transcripts identified in prior studies conducted on fresh plasma samples from patients with AMI [23, 24]. When compared to AMI, a lower number of exosomal miRNAs was DE in CS (Table 3). Several important variables likely factor into the detection of fewer DE transcripts in CS, as explained below, but the results of these investigations are supportive of the premise that circulating exosomal miRNA expression patterns could be used to discriminate inflammatory and ischemic myocardial damage in the clinical setting.

Why should we focus on circulating exosome-derived miRNA for biomarker discovery? One theoretical advantage of exosome-derived genetic biomarkers is their stability, which is a product of the protection conferred by the exosomal membrane against circulating RNase enzymes [25]. Our data strongly supports this paradigm based upon the fact that some of the TIMI II AMI samples have been in storage for over 30 years, yet the quality and quantity of miRNA in these ancient exosomes was comparable to the relatively new GRADS CS samples collected within the past 5–10 years (S1–S3 and S5 Figs). Another compelling advantage of exosomal biomarkers rests upon the premise that the contents of the circulating exosomes differ from other components of the blood in that they are derived directly from diseased or disease-affected cells. This concept is supported by investigations showing distinct miRNA populations in exosomes compared to exosome-depleted plasma [25], and other studies confirming regulation of miRNA expression patterns in exosomes by diseased human tissues [26]. Although we did not confirm a myocardial source of the circulating exosome-derived miRNA in this study, prior publications have documented simultaneous elevated expression of miRs-1, -208b and -499 in myocardium and circulating exosomes in the context of acute myocardial infarction in humans [27], and miR-133 is expressed in cardiac and skeletal muscle [28]. Thus, at least some of the miRNAs identified in this study are from diseased myocardium.

This study shows a particularly strong exosomal miRNA signal in AMI compared to CS. In addition, the AMI signal is remarkably consistent with results of prior studies that focused on plasma or tissue miRNA. We identified 47 exosomal miRNAs having increased concentrations in AMI compared to CS serum samples (Table 3). Of these, 11 miRNA transcripts were validated by qRT-PCR (Figs 5 and 6), and 8 of these 11 have been previously linked to human AMI {miR-1 [23]; miR-133 [23, 24]; miR-146a [28, 29]; miR-208b [23]; miR-335 [30]; miR-423 [24]; miR-483 [31] and miR-499 [27]}. In contrast, only 5 miRNAs showed a significantly increased concentration in CS when compared to AMI serum samples (Table 3), of which 3 have been linked to myocardial disease {miR-144 (validated, Fig 6) [32, 33], miR-624a and miR-624b [34, 35]}.

There are several possible explanations for the weaker circulating exosomal miRNA signal observed in the context of CS compared to AMI. The most significant variable being the diverse phenotypes of CS, typically presenting with one or more of the following disease manifestations: 1) active myocardial (e.g., granulomatous) inflammation; 2) chronic myocardial fibrosis or remodeling, and 3) disease localized to the electrical conduction pathways within the heart. Another factor that likely contributes to increases in the circulating exosomal signal during AMI is the rapid and dramatic onset of myocardial damage induced by acute ischemia involving large portions of the heart. One of the limitations of this study was the inability to accurately classify the CS patients based upon these very different myocardial pathological features. For instance, for the ACCESS study conducted in the late 1990’s, CS was classified based upon clinical features ranging from cardiac arrhythmias to heart failure. There was no objective way to characterize the specific myocardial disease features due to the lack of advanced myocardial imaging technology at the time of the study. For the GRADS study, conducted more recently, the detection of cardiac sarcoidosis was not standardized. In some cases, CS detection was based upon MRI with late gadolinium enhancement, which most reliably detects chronic fibrosis or remodeling. In other GRADS cases, CS was identified by FDG-18 PET scan, which reliably detects active cardiac inflammation as opposed to fibrosis or remodeling. It is reasonable to speculate that there are different circulating exosomal miRNA patterns in those with active cardiac inflammation compared to patients with established chronic cardiac fibrosis. Furthermore, an inflammatory biomarker would have greater clinical utility in terms of guiding immune suppression therapies for CS. Thus, future studies should focus on the identification of CS biomarkers that are specific for the detection of acute myocarditis.

Another limitation of the study relates to the processing of the samples, particularly as relates to comparing results from exosomes derived from serum versus plasma. Serum and plasma extracellular miRNA profiles are different, and serum has fewer miRNA detected overall, as we have demonstrated in S5 and S9 Figs, likely relating to cell stress and loss of exosomes during clot formation [36, 37]. Plasma is preferred for this reason, and may explain why common CS miRNA were not identified when comparing CS to controls (plasma samples) and CS to AMI (serum samples).

## Conclusions

The detection of active myocardial inflammation in sarcoidosis patients is a challenge. The clinical presentation can resemble cardiac ischemia [7–10], and definitive diagnosis of CS requires that cardiac ischemia be excluded [38, 39]. Circulating exosomal miRNA show promise as biomarkers that can distinguish CS from AMI and with further development perhaps other inflammatory cardiomyopathies. Future studies focusing on CS patients with radiographic evidence of active inflammation or chronic fibrosis are likely to yield more reliable circulating exosomal miRNA biomarkers for the detection of CS. Since circulating extracellular miRNAs are quite stable [40], they provide promise for improved CS detection and may prove useful for distinguishing other severe sarcoidosis phenotypes (e.g., neurosarcoidosis).

## Supporting information

S1 Fig Representative exosomal spectra following isolation in each study group sample. Representative results from NanoSight analysis, following the isolation of the exosomal fraction from the plasma/serum study samples, showing similar exosomal (particle) spectral profile, size distribution and concentration.
(TIF)

S2 Fig High degree of similarity in the exosomal distribution and yield as isolated from samples in each study group. Group results from NanoSight analyses of the exosomes isolated from the plasma/serum study samples demonstrating nearly identical exosomal (particle) mean size at the distribution peak (A) along with their total concentration yield (B), despite the relatively extensive and varying age of the samples. Results indicated that the exosomes remained remarkably and similarly intact while frozen over time and following isolation from their source samples.
(TIF)

S3 Fig Further evidence demonstrating the similarity in exosomal properties isolated from samples in each study group. Despite the relatively extensive and varying age of the samples, NanoSight analyses of exosomes isolated from the plasma/serum demonstrated nearly identical dynamic light scatter results.
(TIF)

S4 Fig Evaluation of extracted exosomal miRNA demonstrates consistent quality among the samples in each study group. Quality assessment of exosomal miRNA isolated from the plasma/serum samples of the ACCESS/TIMI II/GRADS studies following next generation sequencing analyses. Principle component analysis plot shows that there was no obvious batch effect across the samples. Three samples looked quite different from the others (orange circle) and were ultimately eliminated from the final differential expression analyses.
(TIF)

S5 Fig Differential number of the extracted exosomal miRNAs detected among the samples in each study group. Distribution of detected exosomal miRNAs extracted from the plasma/serum samples. Generally, plasma samples had a higher number detected exosomal miRNAs than serum. Within the plasma or serum samples, the sarcoid group had a lower number of detected exosomal miRNAs than the comparative control or acute myocardial infarction (AMI) groups, respectively.
(TIF)

S6 Fig Differential expression of the extracted exosomal miRNAs between the cardiac sarcoidosis and control plasma study samples. (Top) A volcano plot representing differential expression (DE) analysis of exosomal miRNAs extracted from the cardiac sarcoid and control plasma study samples after removal of samples with a low read depth (<10^6^ reads). Wherein the Y-axis corresponds to transcripts with high statistical significance (-log 10 of p-value), and the X-axis corresponds with fold-change of gene expression (log base 2) generated from the same data set. DE transcripts on the upper left side of the plot have strong statistical significance with relatively low expression; whereas, transcripts on the upper right are more highly expressed with strong statistical significance. The blue lines correspond with a fold-change of 1.5 and p-value cutoff of 0.05; red dashed lines as marked. (Bottom) DE analyses demonstrated a number of up- and down-regulated transcripts at two levels of significance.
(TIF)

S7 Fig Validation of selected differentially expressed miRNA transcripts between cardiac sarcoidosis and control plasma exosomal samples. Additional miRNA transcripts [(A) miR-889-3p and (B) miR-376-3p], determined to be differentially expressed by next generation sequencing when comparing the cardiac sarcoidosis (CS) and control groups, were confirmed by qRT-PCR in the plasma exosomal validation samples (*p < 0.05).
(TIF)

S8 Fig Differential expression of the extracted exosomal miRNAs between the cardiac sarcoidosis and acute myocardial infarction serum study samples. (Top) A volcano plot representing differential expression (DE) analysis of exosomal miRNAs extracted from the cardiac sarcoid and acute myocardial infarction (AMI) serum study samples after removal of samples with a low read depth (<10^6^ reads). Wherein the Y-axis corresponds to transcripts with high statistical significance (-log 10 of p-value), and the X-axis corresponds with fold-change of gene expression (log base 2) generated from the same data set. DE transcripts on the upper left side of the plot have strong statistical significance with relatively low expression; whereas, transcripts on the upper right are more highly expressed with strong statistical significance. The blue lines correspond with a fold-change of 1.5 and p-value cutoff of 0.05; red dashed lines as marked. (Bottom) DE analyses demonstrated a number of up- and down-regulated transcripts at two levels of significance.
(TIF)

S9 Fig Differential expression of the extracted exosomal miRNAs between the cardiac sarcoidosis plasma and serum study samples. (Top) A volcano plot representing differential expression (DE) analysis of exosomal miRNAs extracted from the cardiac sarcoid plasma and serum study samples after removal of samples with a low read depth (<10^6^ reads). Wherein the Y-axis corresponds to transcripts with high statistical significance (-log 10 of p-value), and the X-axis corresponds with fold-change of gene expression (log base 2) generated from the same data set. DE transcripts on the upper left side of the plot have strong statistical significance with relatively low expression; whereas, transcripts on the upper right are more highly expressed with strong statistical significance. The blue lines correspond with a fold-change of 1.5 and p-value cutoff of 0.05; red dashed lines as marked. (Bottom) DE analyses demonstrated a number of up- and down-regulated transcripts at two levels of significance.
(TIF)
