# Supplementary figures and images for: Circulating exosomal microRNA expression patterns distinguish cardiac sarcoidosis from myocardial ischemia
Source: PLoS One. 2021 Jan 26;16(1):e0246083. doi: 10.1371/journal.pone.0246083 (PMC7837479; doi:10.1371/journal.pone.0246083)

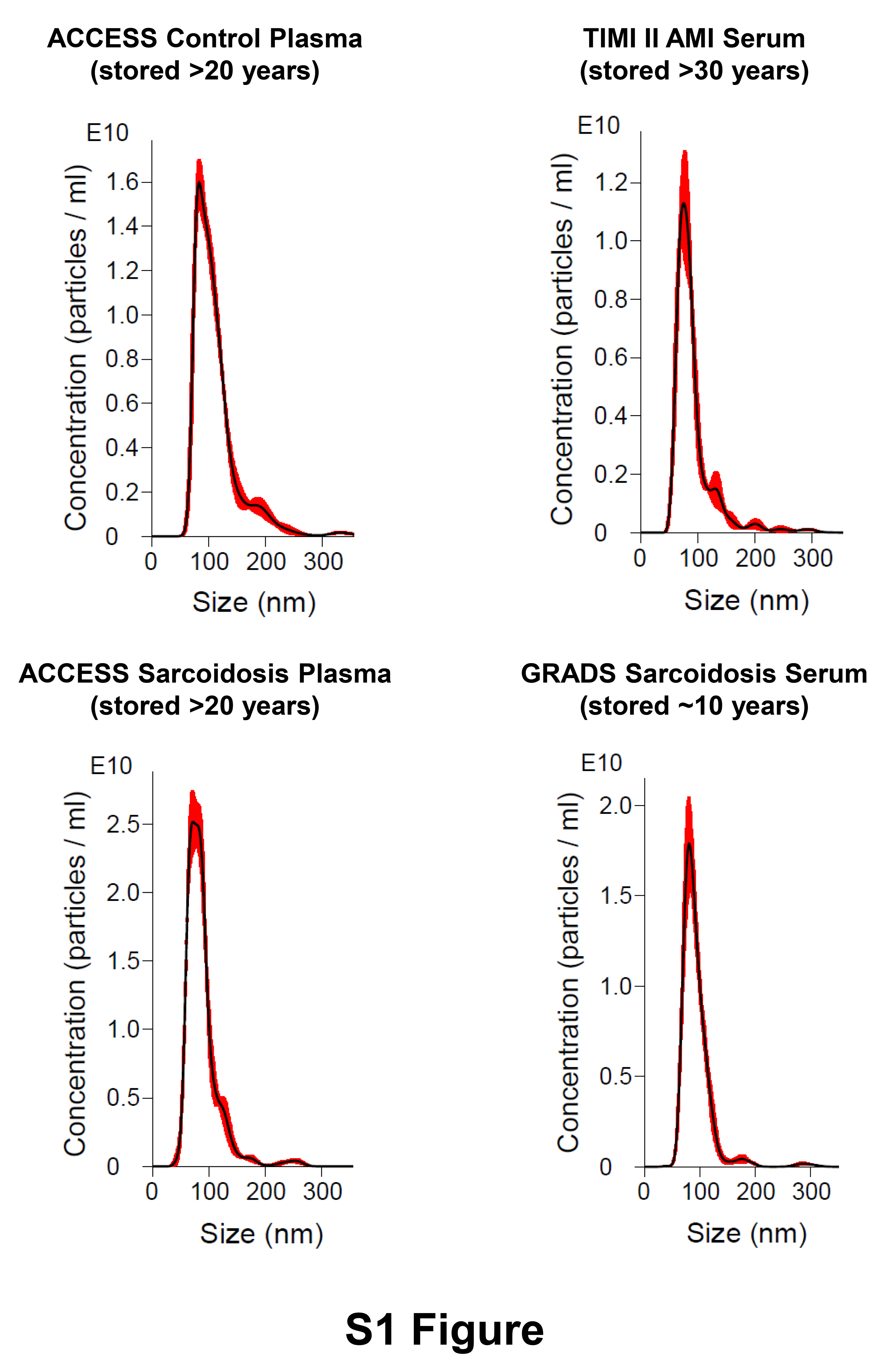

Supplement: S1 Fig — Representative results from NanoSight analysis, following the isolation of the exosomal fraction from the plasma/serum study samples, showing similar exosomal (particle) spectral profile, size distribution and concentration. (TIF) [file pone.0246083.s001.tif]

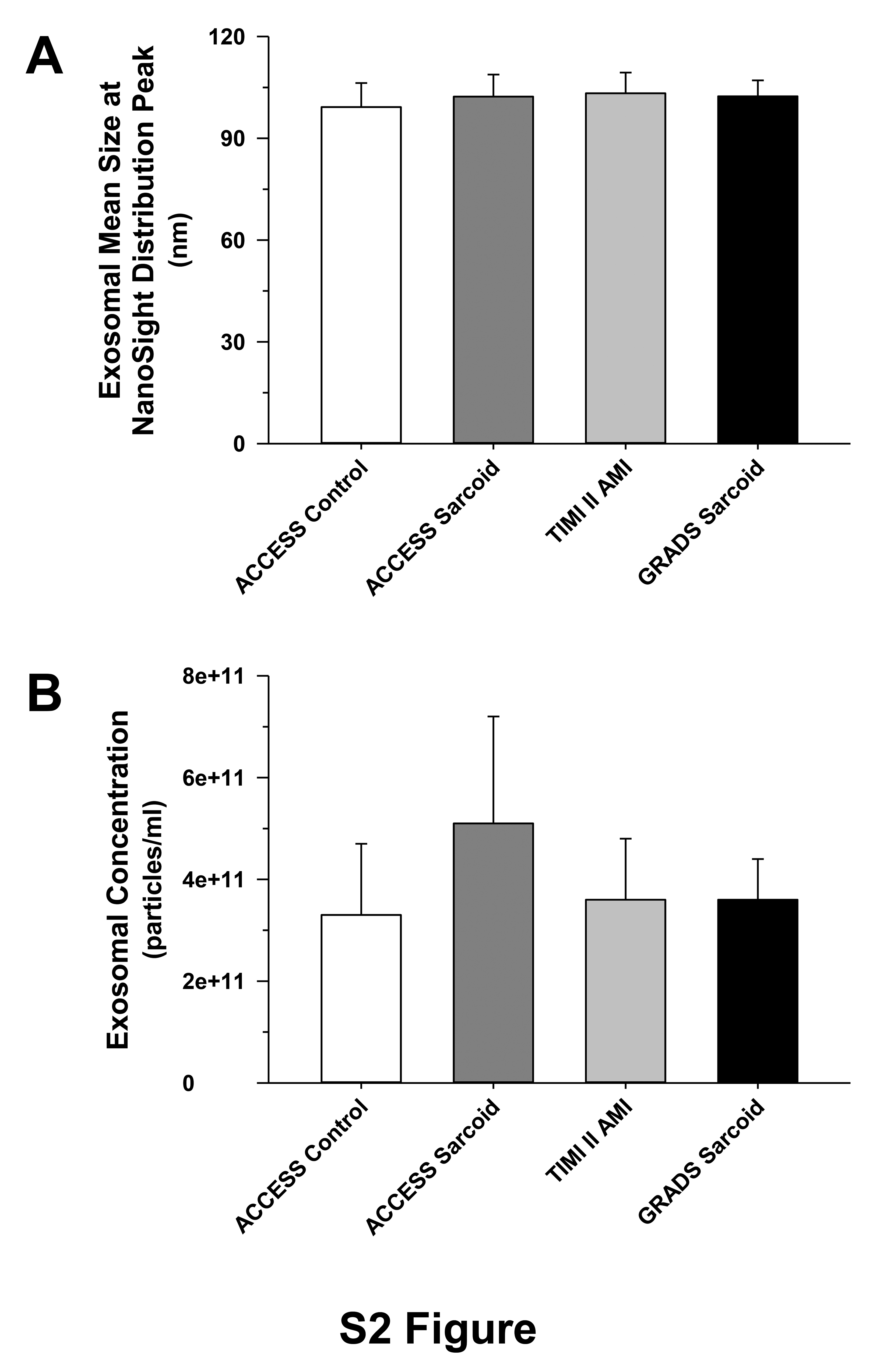

Supplement: S2 Fig — Group results from NanoSight analyses of the exosomes isolated from the plasma/serum study samples demonstrating nearly identical exosomal (particle) mean size at the distribution peak (A) along with their total concentration yield (B), despite the relatively extensive and varying age of the samples. Results indicated that the exosomes remained remarkably and similarly intact while frozen over time and following isolation from their source samples. (TIF) [file pone.0246083.s002.tif]

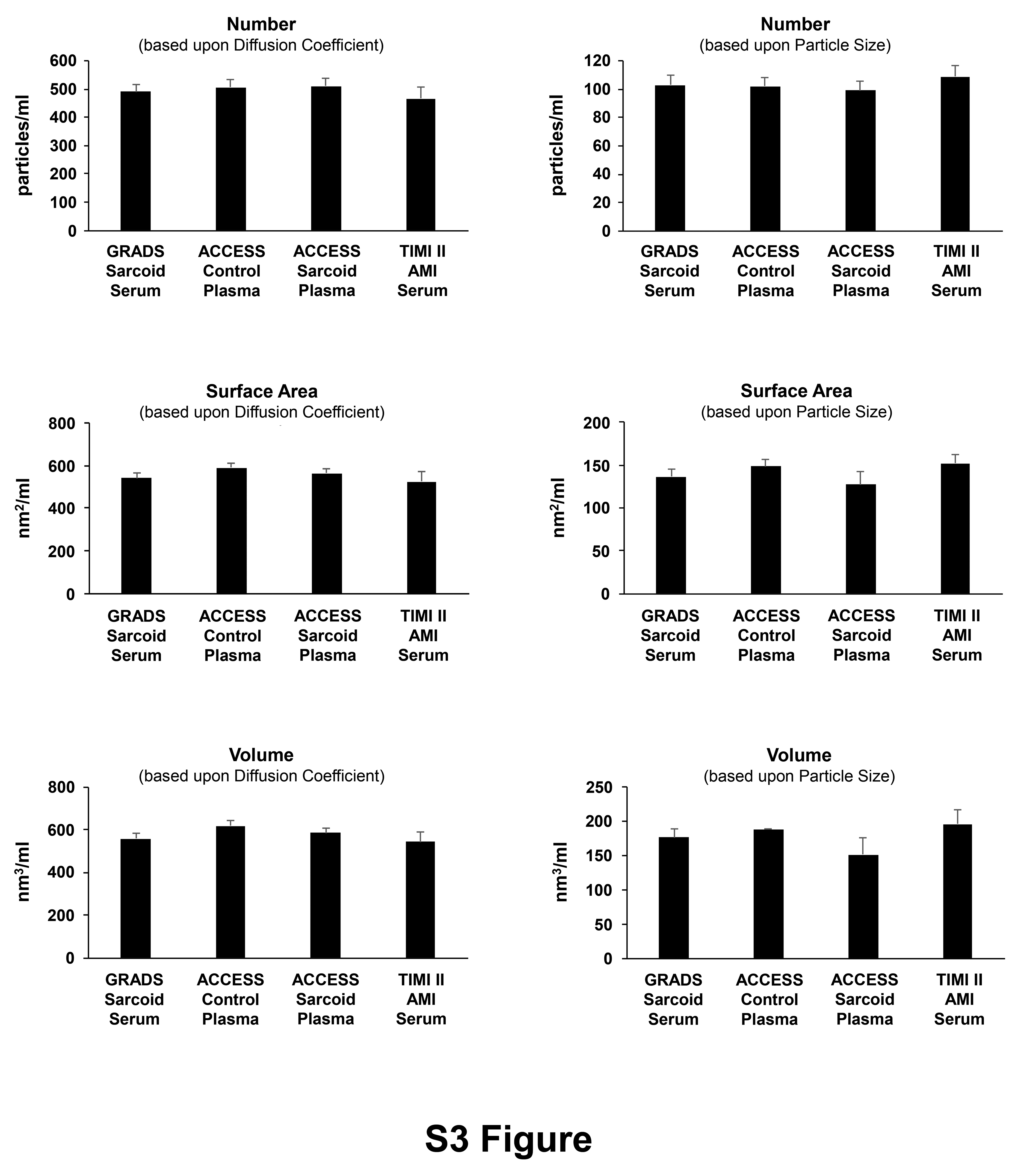

Supplement: S3 Fig — Despite the relatively extensive and varying age of the samples, NanoSight analyses of exosomes isolated from the plasma/serum demonstrated nearly identical dynamic light scatter results. (TIF) [file pone.0246083.s003.tif]

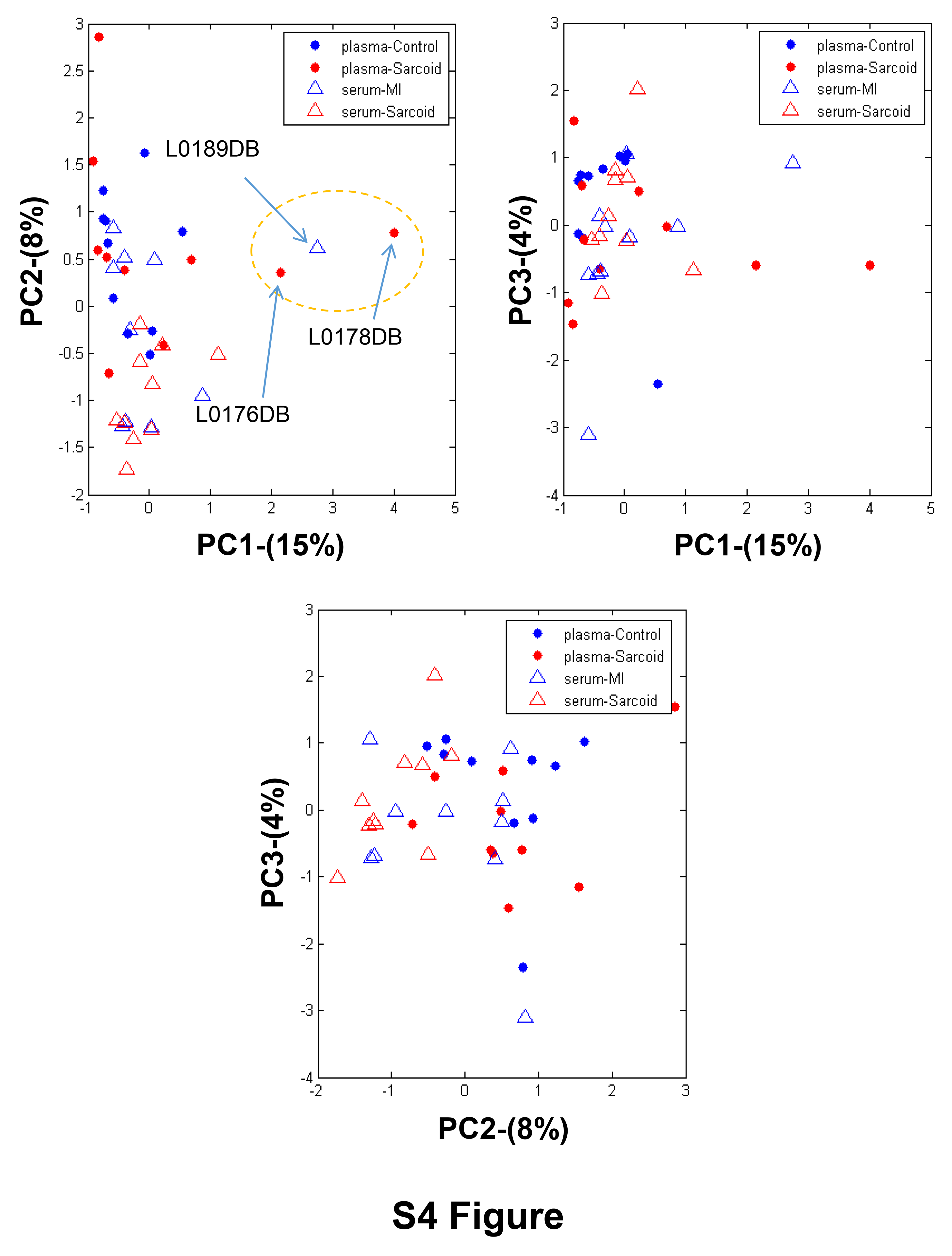

Supplement: S4 Fig — Quality assessment of exosomal miRNA isolated from the plasma/serum samples of the ACCESS/TIMI II/GRADS studies following next generation sequencing analyses. Principle component analysis plot shows that there was no obvious batch effect across the samples. Three samples looked quite different from the others (orange circle) and were ultimately eliminated from the final differential expression analyses. (TIF) [file pone.0246083.s004.tif]

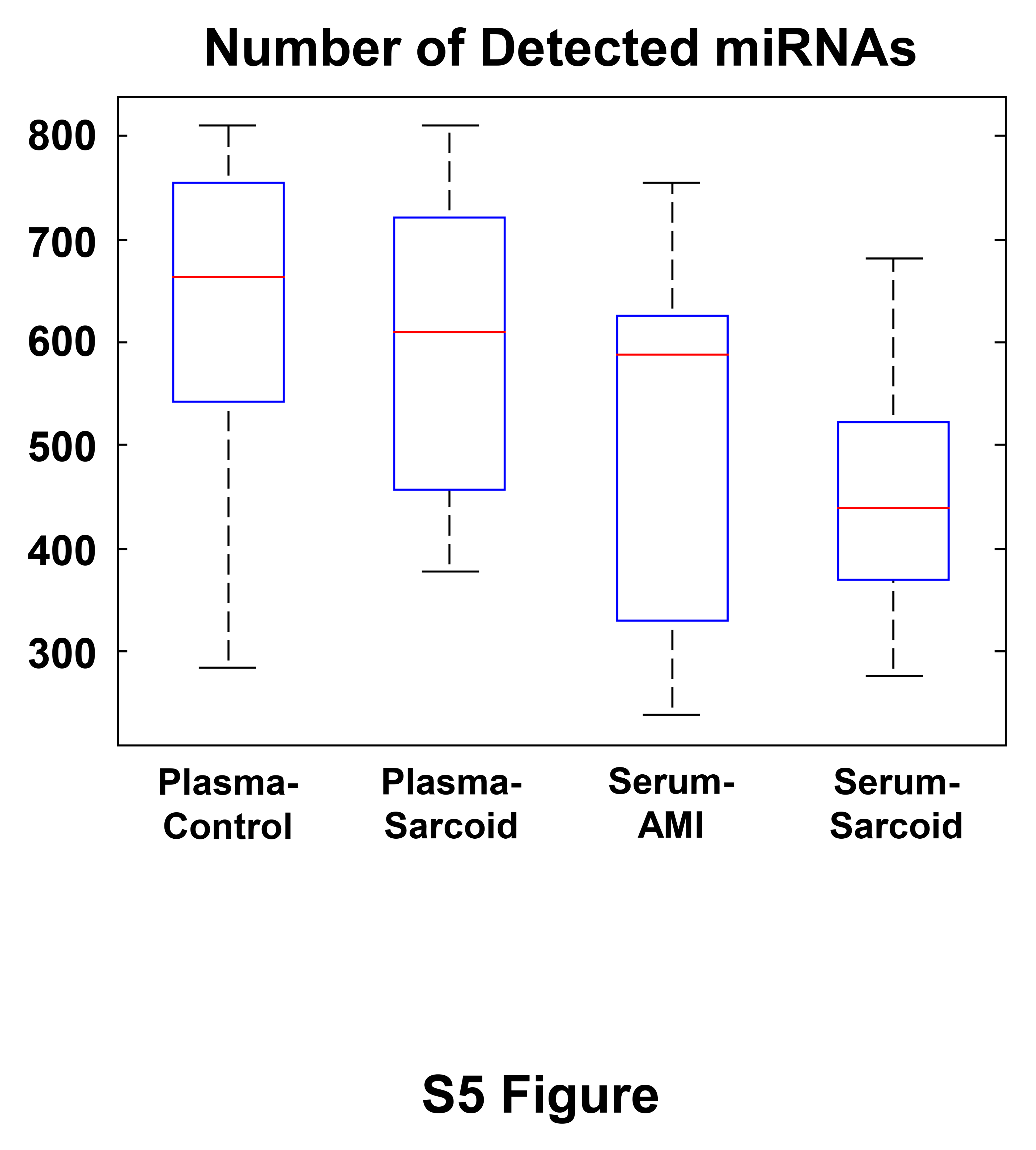

Supplement: S5 Fig — Distribution of detected exosomal miRNAs extracted from the plasma/serum samples. Generally, plasma samples had a higher number detected exosomal miRNAs than serum. Within the plasma or serum samples, the sarcoid group had a lower number of detected exosomal miRNAs than the comparative control or acute myocardial infarction (AMI) groups, respectively. (TIF) [file pone.0246083.s005.tif]

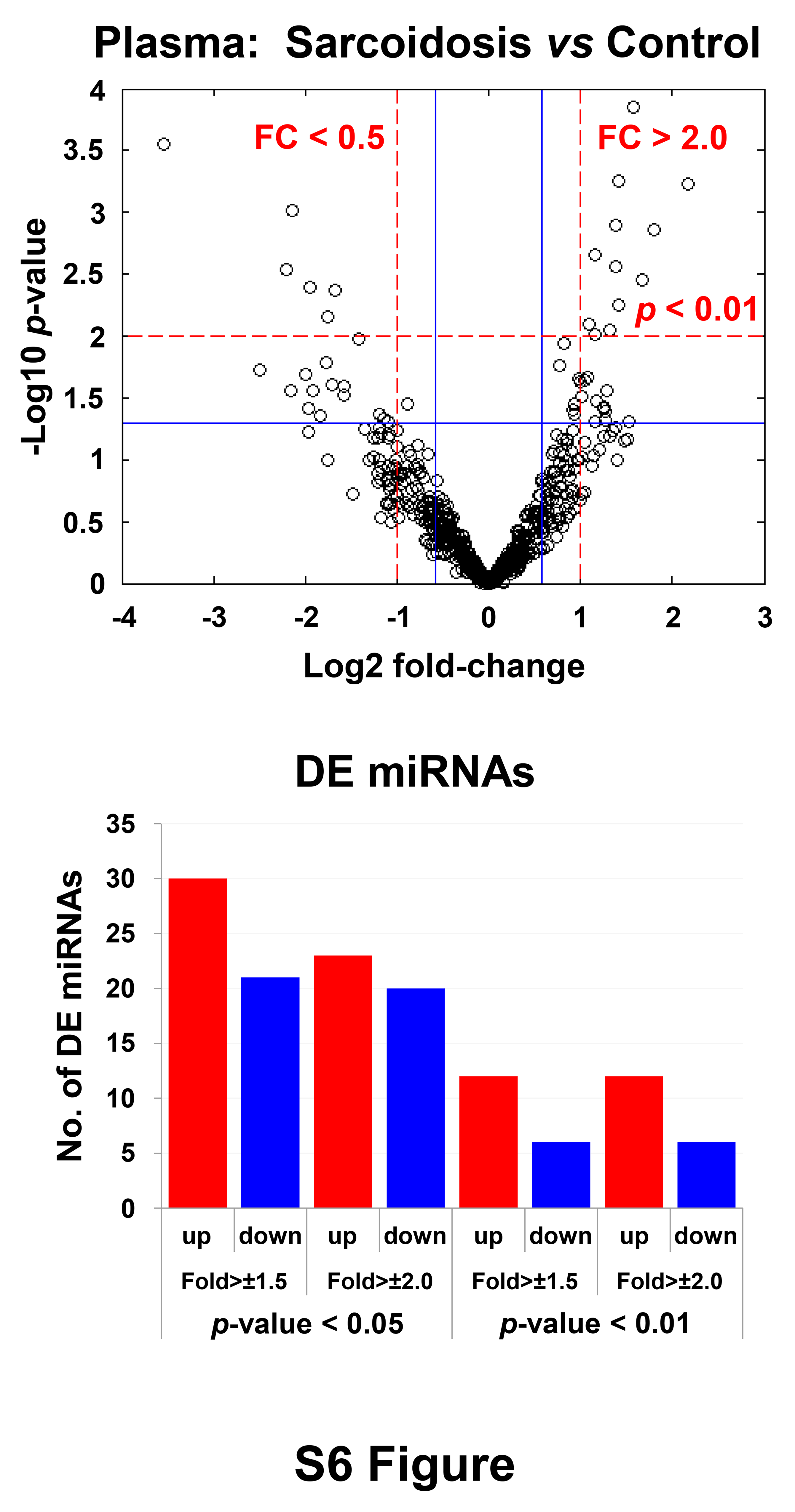

Supplement: S6 Fig — (Top) A volcano plot representing differential expression (DE) analysis of exosomal miRNAs extracted from the cardiac sarcoid and control plasma study samples after removal of samples with a low read depth (<106 reads). Wherein the Y-axis corresponds to transcripts with high statistical significance (-log 10 of p-value), and the X-axis corresponds with fold-change of gene expression (log base 2) generated from the same data set. DE transcripts on the upper left side of the plot have strong statistical significance with relatively low expression; whereas, transcripts on the upper right are more highly expressed with strong statistical significance. The blue lines correspond with a fold-change of 1.5 and p-value cutoff of 0.05; red dashed lines as marked. (Bottom) DE analyses demonstrated a number of up- and down-regulated transcripts at two levels of significance. (TIF) [file pone.0246083.s006.tif]

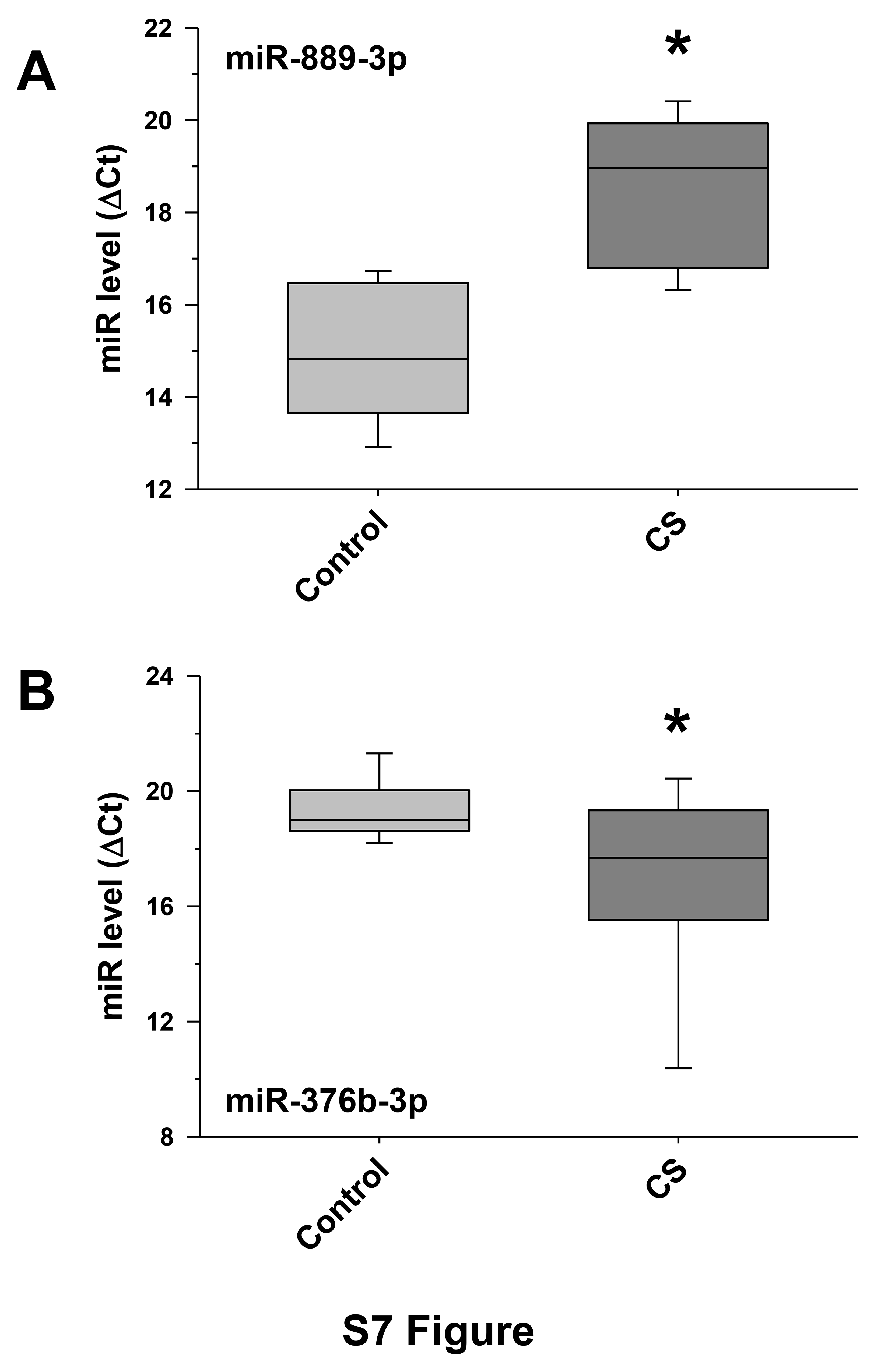

Supplement: S7 Fig — Additional miRNA transcripts [(A) miR-889-3p and (B) miR-376-3p], determined to be differentially expressed by next generation sequencing when comparing the cardiac sarcoidosis (CS) and control groups, were confirmed by qRT-PCR in the plasma exosomal validation samples (*p < 0.05). (TIF) [file pone.0246083.s007.tif]

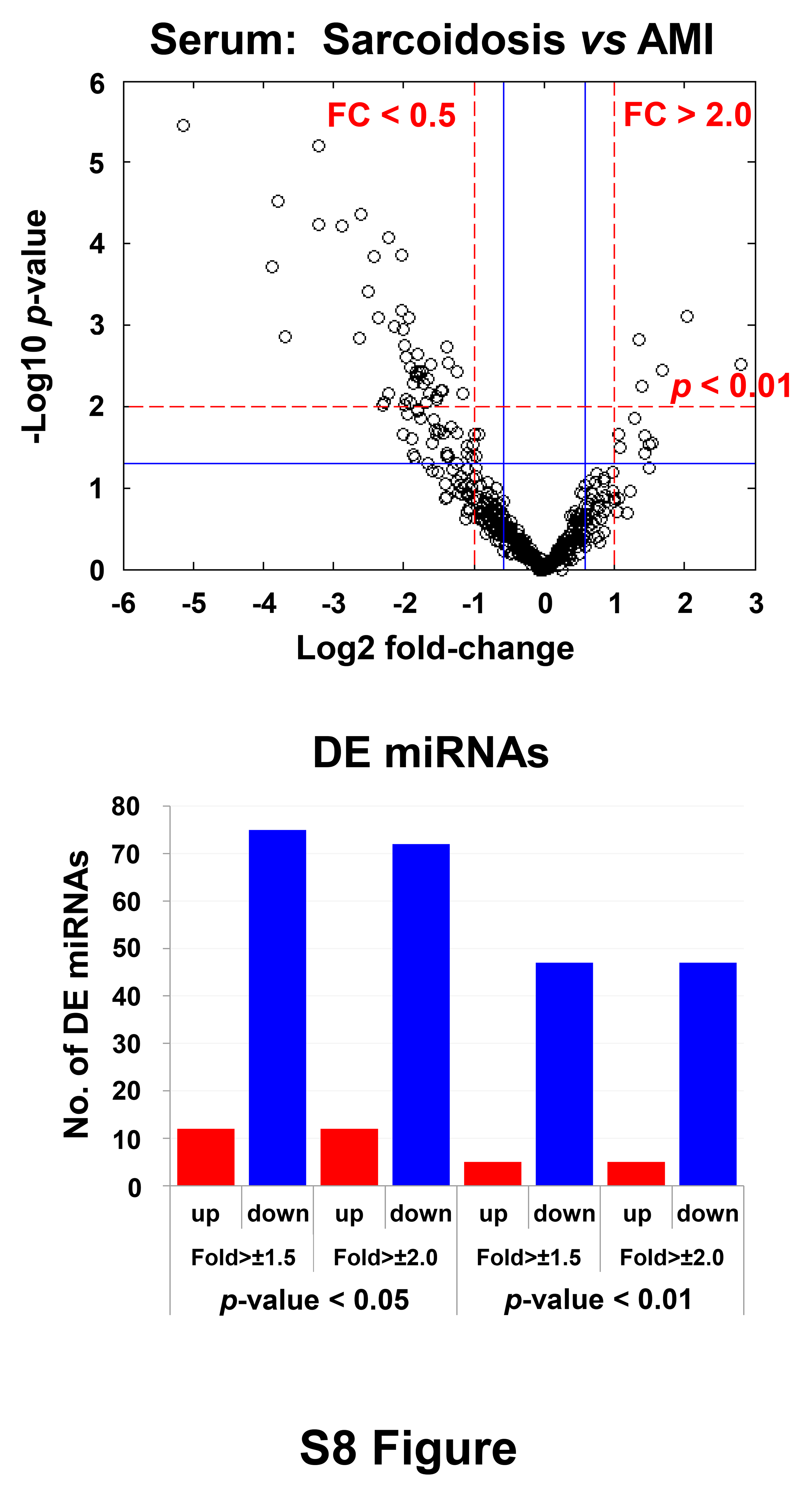

Supplement: S8 Fig — (Top) A volcano plot representing differential expression (DE) analysis of exosomal miRNAs extracted from the cardiac sarcoid and acute myocardial infarction (AMI) serum study samples after removal of samples with a low read depth (<106 reads). Wherein the Y-axis corresponds to transcripts with high statistical significance (-log 10 of p-value), and the X-axis corresponds with fold-change of gene expression (log base 2) generated from the same data set. DE transcripts on the upper left side of the plot have strong statistical significance with relatively low expression; whereas, transcripts on the upper right are more highly expressed with strong statistical significance. The blue lines correspond with a fold-change of 1.5 and p-value cutoff of 0.05; red dashed lines as marked. (Bottom) DE analyses demonstrated a number of up- and down-regulated transcripts at two levels of significance. (TIF) [file pone.0246083.s008.tif]

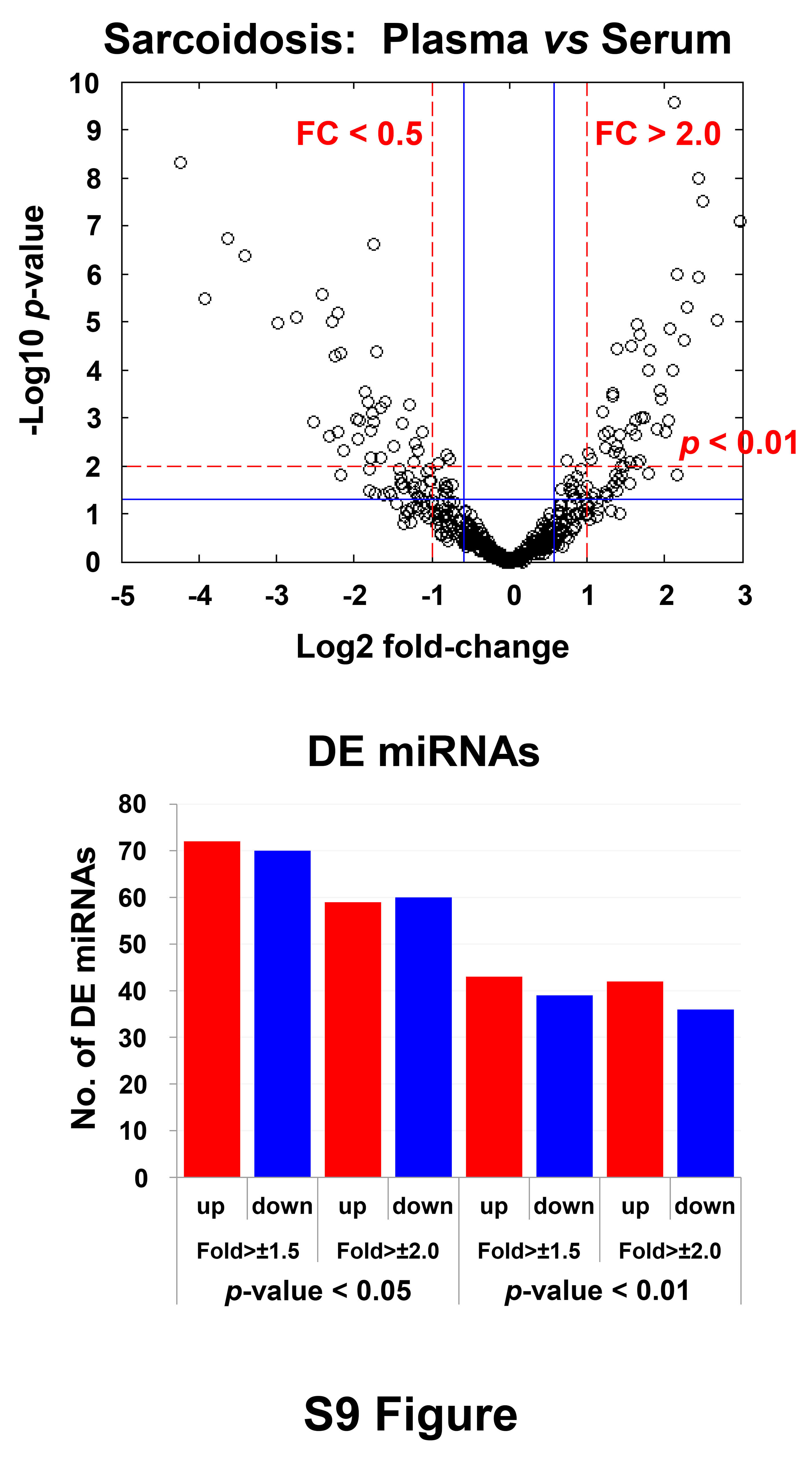

Supplement: S9 Fig — (Top) A volcano plot representing differential expression (DE) analysis of exosomal miRNAs extracted from the cardiac sarcoid plasma and serum study samples after removal of samples with a low read depth (<106 reads). Wherein the Y-axis corresponds to transcripts with high statistical significance (-log 10 of p-value), and the X-axis corresponds with fold-change of gene expression (log base 2) generated from the same data set. DE transcripts on the upper left side of the plot have strong statistical significance with relatively low expression; whereas, transcripts on the upper right are more highly expressed with strong statistical significance. The blue lines correspond with a fold-change of 1.5 and p-value cutoff of 0.05; red dashed lines as marked. (Bottom) DE analyses demonstrated a number of up- and down-regulated transcripts at two levels of significance. (TIF) [file pone.0246083.s009.tif]
